# Supplementary material for: Biocompatible PVDF Nanofibers with Embedded Magnetite Nanodiscs Enable Wireless Magnetoelectric Stimulation in Premotor Cortex
Source: Adv Healthc Mater. 2025 Aug 7;14(32):e03082. doi: 10.1002/adhm.202503082 (PMC12716173; doi:10.1002/adhm.202503082)
Supplement: Supplementary file 1 — Supporting Information [file ADHM-14-0-s001.docx]

Supporting Information

BIOCOMPATIBLE PVDF NANOFIBERS WITH EMBEDDED MAGNETITE NANODISCS ENABLE WIRELESS MAGNETOELECTRIC STIMULATION IN PREMOTOR CORTEX.

Lorenzo Signorelli, Anouk Wolters, Vicente Duran Toro, Jonas Englhard, Mahdieh Shojaei Baghini, Elif Koҫar, Franziska Wasner, Nadine Goldenstein, Hadi Heidari, Julien Bachmann, Sarah Hescham*, Danijela Gregurec*


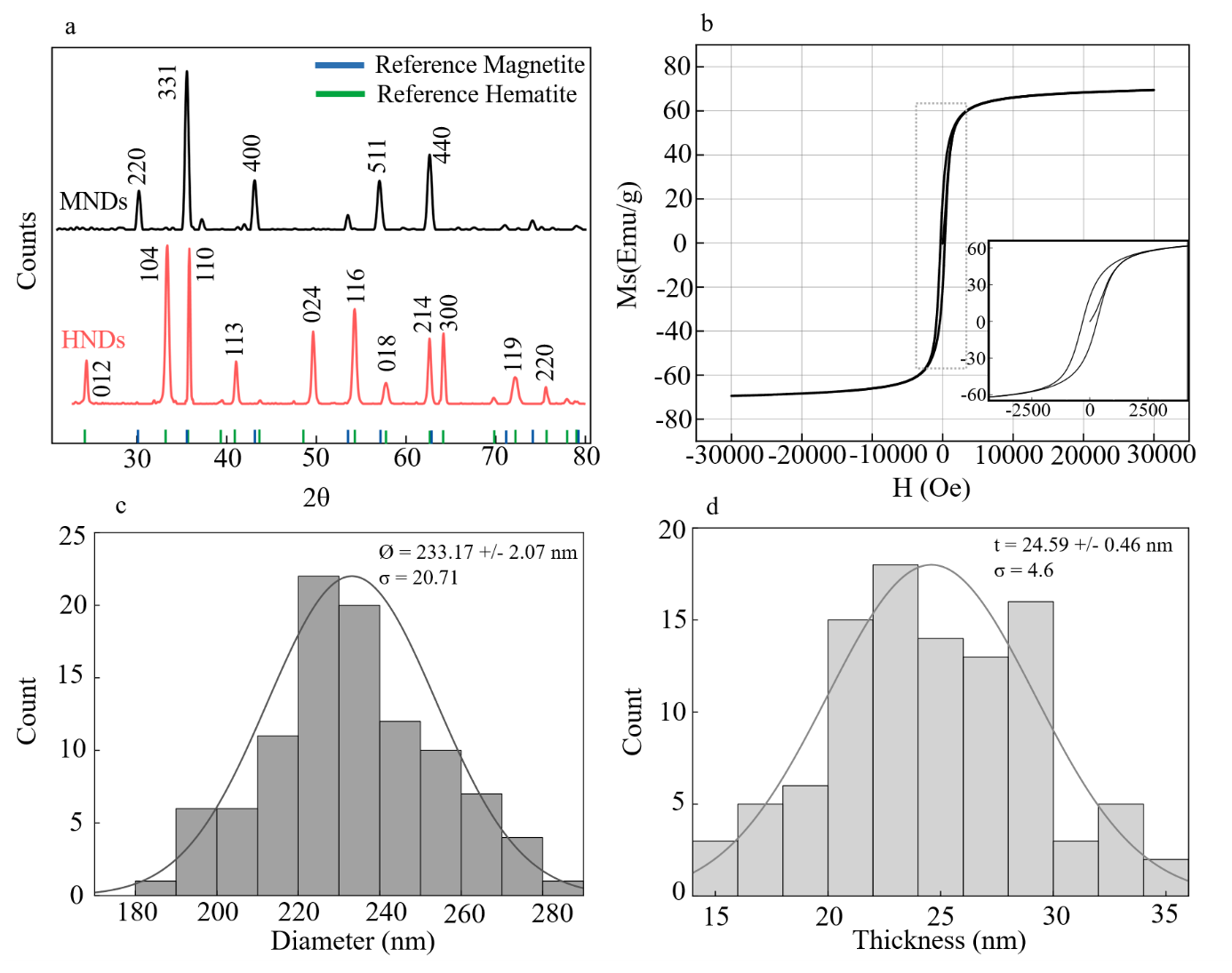


**Figure S1:** Magnetic nanodiscs material characterization a) XRD diffractograms of hematite nanodiscs (HNDs) and mangetite nanodiscs (MNDs) with references included, b) Saturation magnetization of MNDs (expressed as emu per gram of MND), c) Statistical distribution of MND diameter, and d) thickness.

**
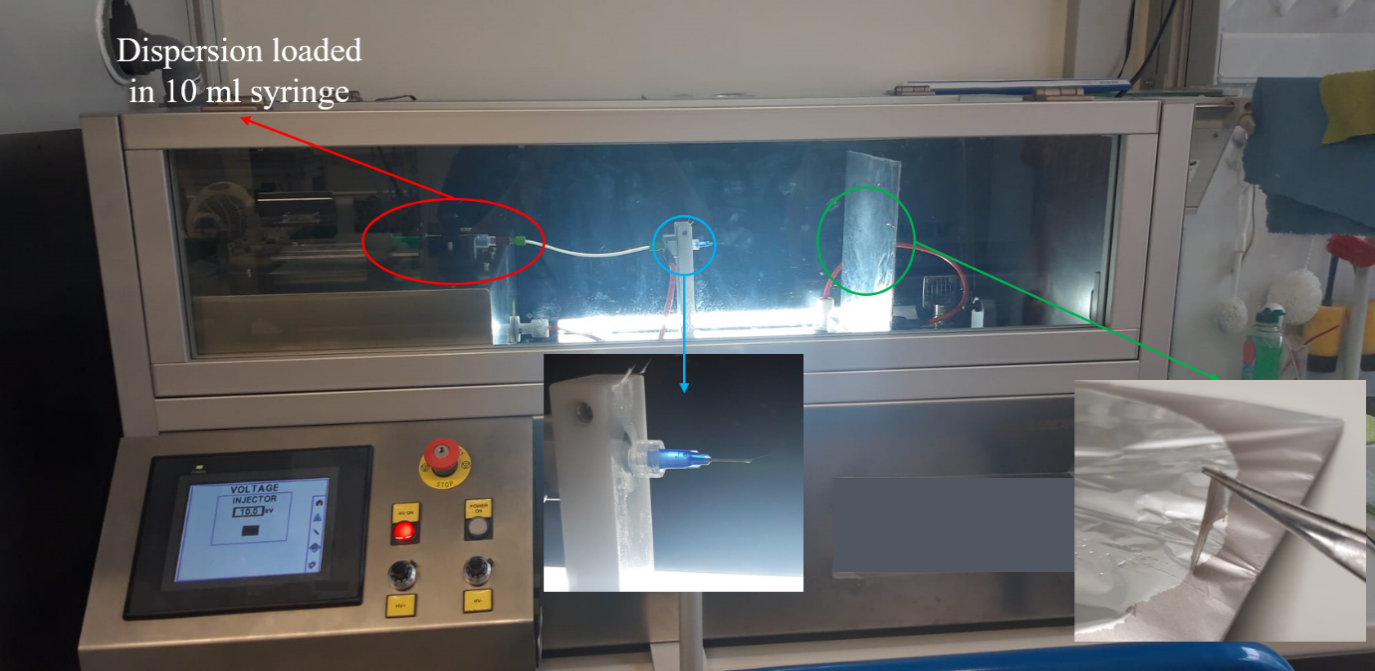
**

**Figure S2:** The dispersion of PVDF and nanodiscs is loaded into a 10 mL syringe and pushed through to a needle where a high voltage is applied. The extruded dispersion is then drawn toward the collector on the opposite side and stretched into a fiber. The resulting fibers are subsequently collected using tweezers.

**
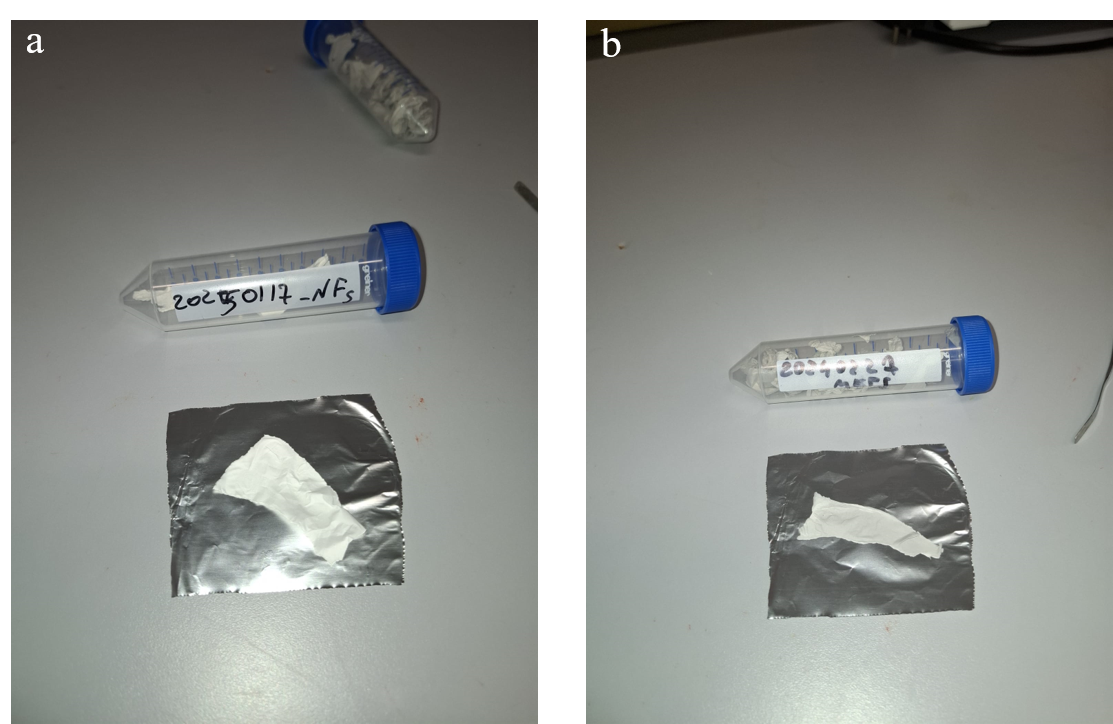
**

**Figure S3:** The fibers produced by electrospinning resemble a fabric; they are therefore very flexible and easy to handle.

**
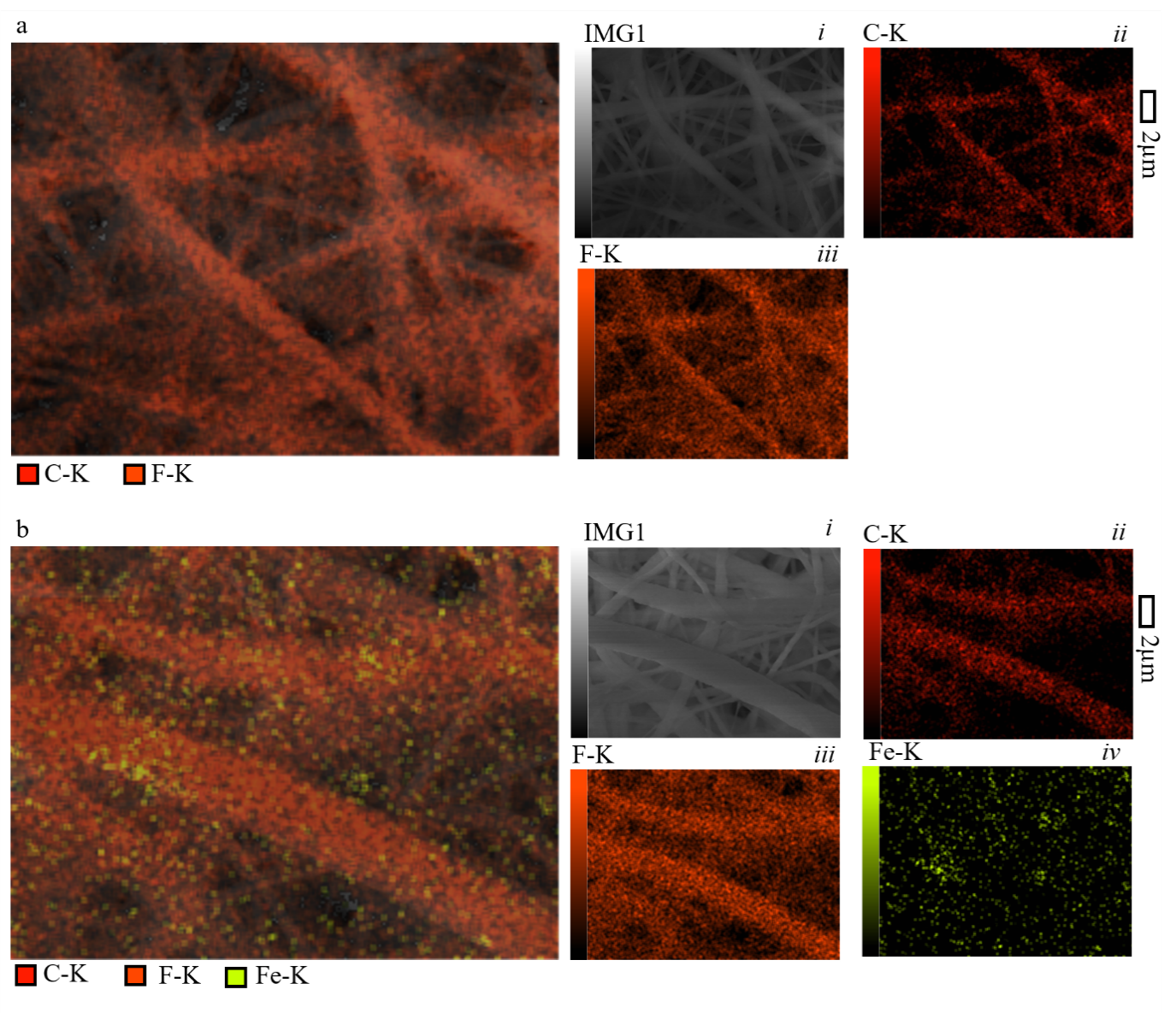
**

**Figure S4:** Elementary analysis by Energy Dispersive X-ray analysis (EDX) of nanofibres shows that: a) in pure PVDF nanofibres (NFs), combined with the corresponding SEM image (*i*), the two constituent elements of PVDF, carbon (*ii*) and fluorine (*iii*) are identified; b) in PVDF fibers enriched with MNDs, alongside the SEM image (*i*), in addition to carbon (*ii*) and fluorine (*iii*), the presence of iron (*iv*) is also detected, confirming the successful incorporation of MNDs.

**
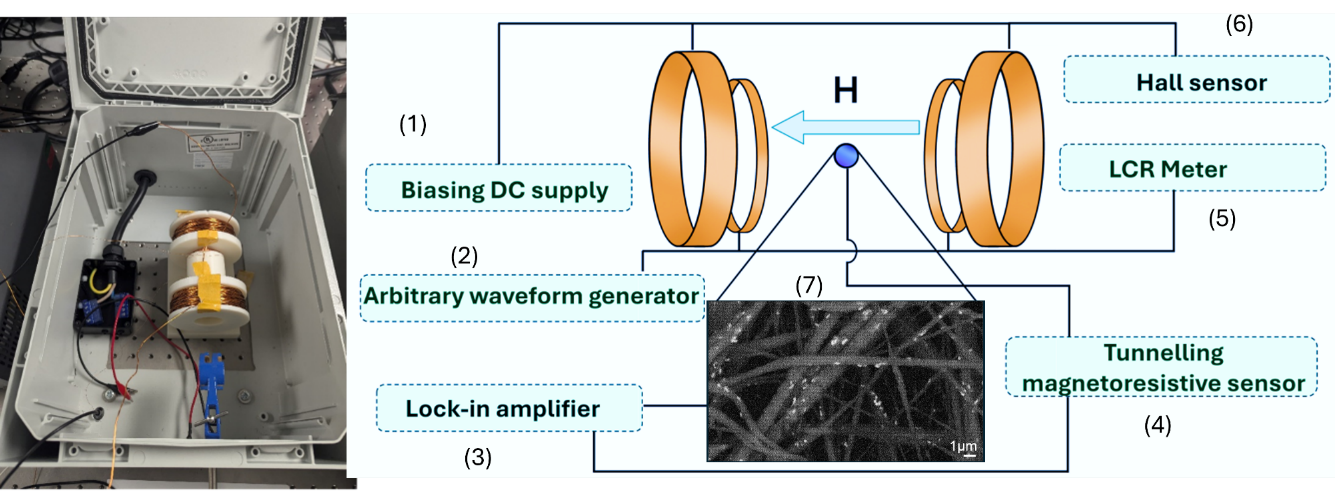
**

**Figure S5:** Setup and schematic diagram of the experimental setup used to characterize the magnetoelectric (ME) response of nanofibers. The setup includes: 1. A DC power supply or permanent magnets that provide a DC magnetic field in the plane of the nanofibers; 2. AC power generator that applies an AC field along the same plane; 3. Lock-in amplifier (Zurich Instruments) records the output voltage variation due to the ME fibers at the set frequency; 4. TMR sensor (Neuranics Ltd) for monitoring the AC magnetic field; 5. LCR meter (Keysight E4980AL) for monitoring the impedance of the AC coil to ensure that the coil functionality is not degraded at higher amplitudes; 6. Hall sensor (TLV493D) to measure the DC magnetic field; 7. SEM micrograph of MEFs

**
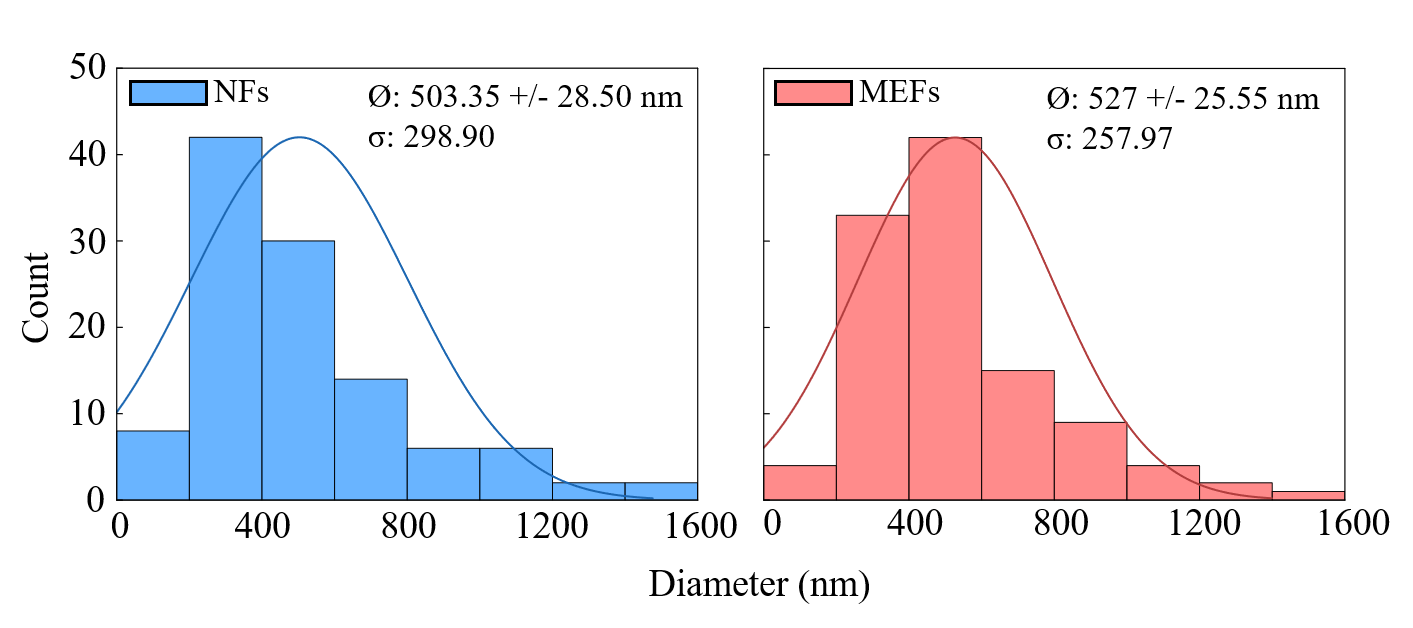
**

**Figure S6:** Statistical distribution of the NFs and MEFs diameter.

**
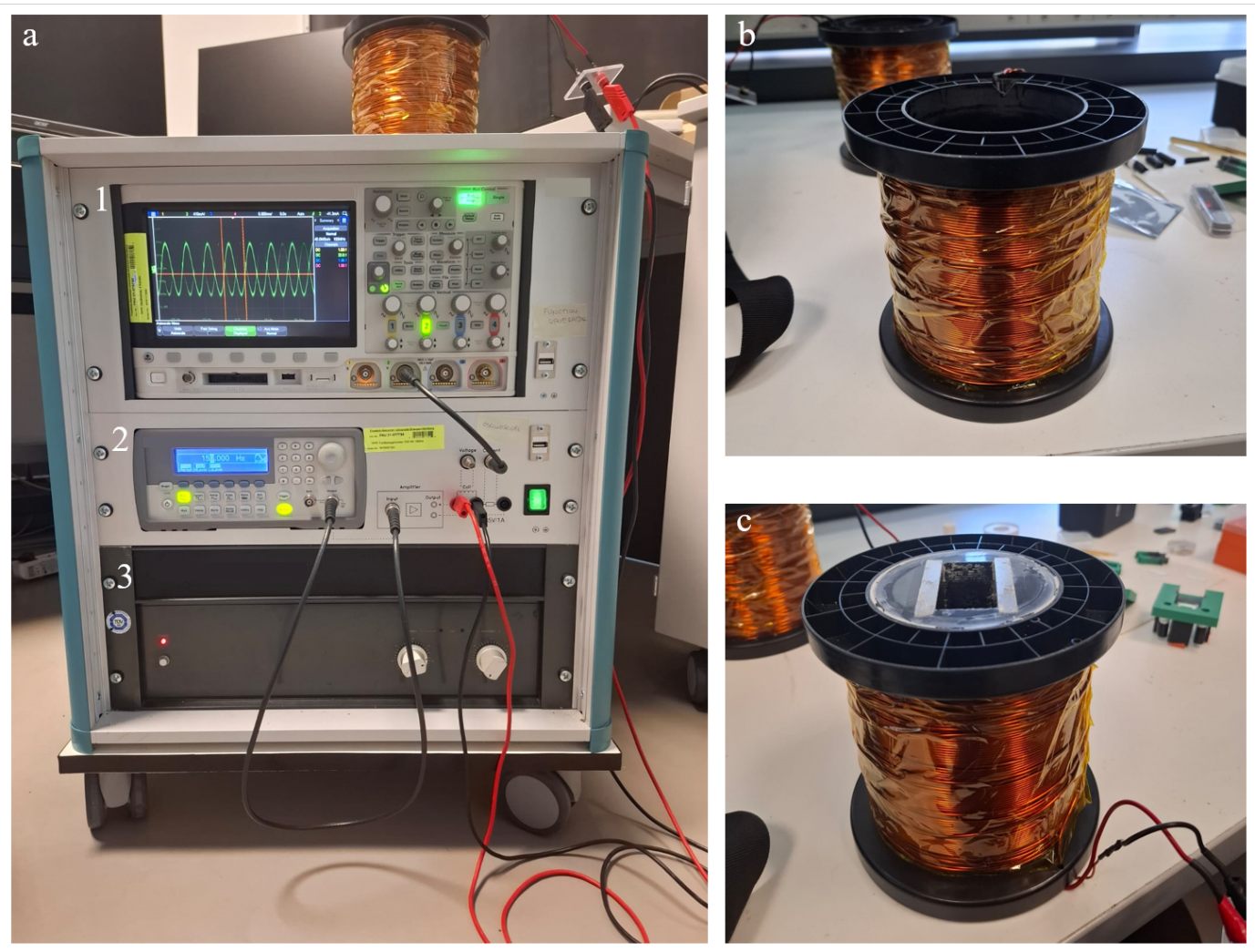
**

**Figure S7:** The setup used for stimulating the fibers during calcium imaging consists of: a) a main unit containing an oscilloscope (1), a wave generator (2), and an amplifier (3); this system is paired with b) a 5-kg copper coil for the magnetomechanical stimulation of the fibers; and for magnetoelectric stimulation, c) a 5-kg copper coil with two fixed neodymium permanent magnets inside, generating a constant magnetic field of 220 mT.

**
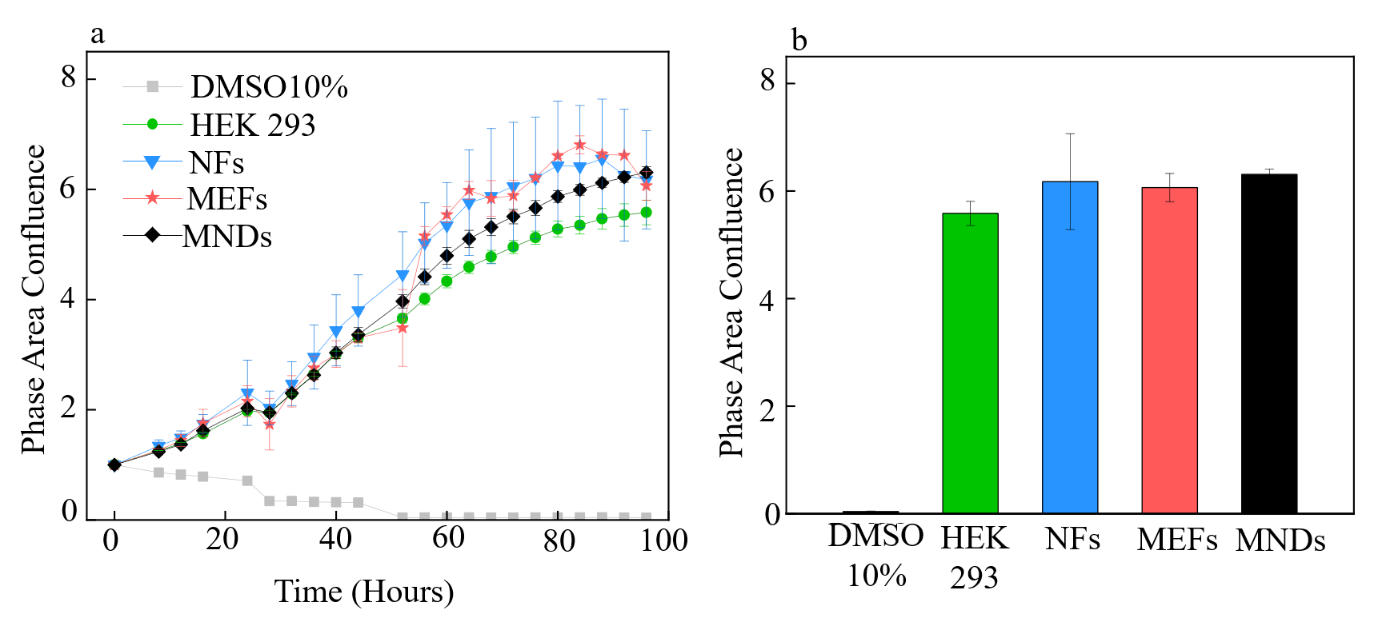
**

**Figure S8:** Phase area confluence of HEK293 cells: a) Variation of phase area confluence over a period of 100 hours in the presence of DMSO as a negative control, HEK293 cells as baseline control, NFs, MEFs, and MNDs; b) Phase area confluence at the end of the 100-hour period


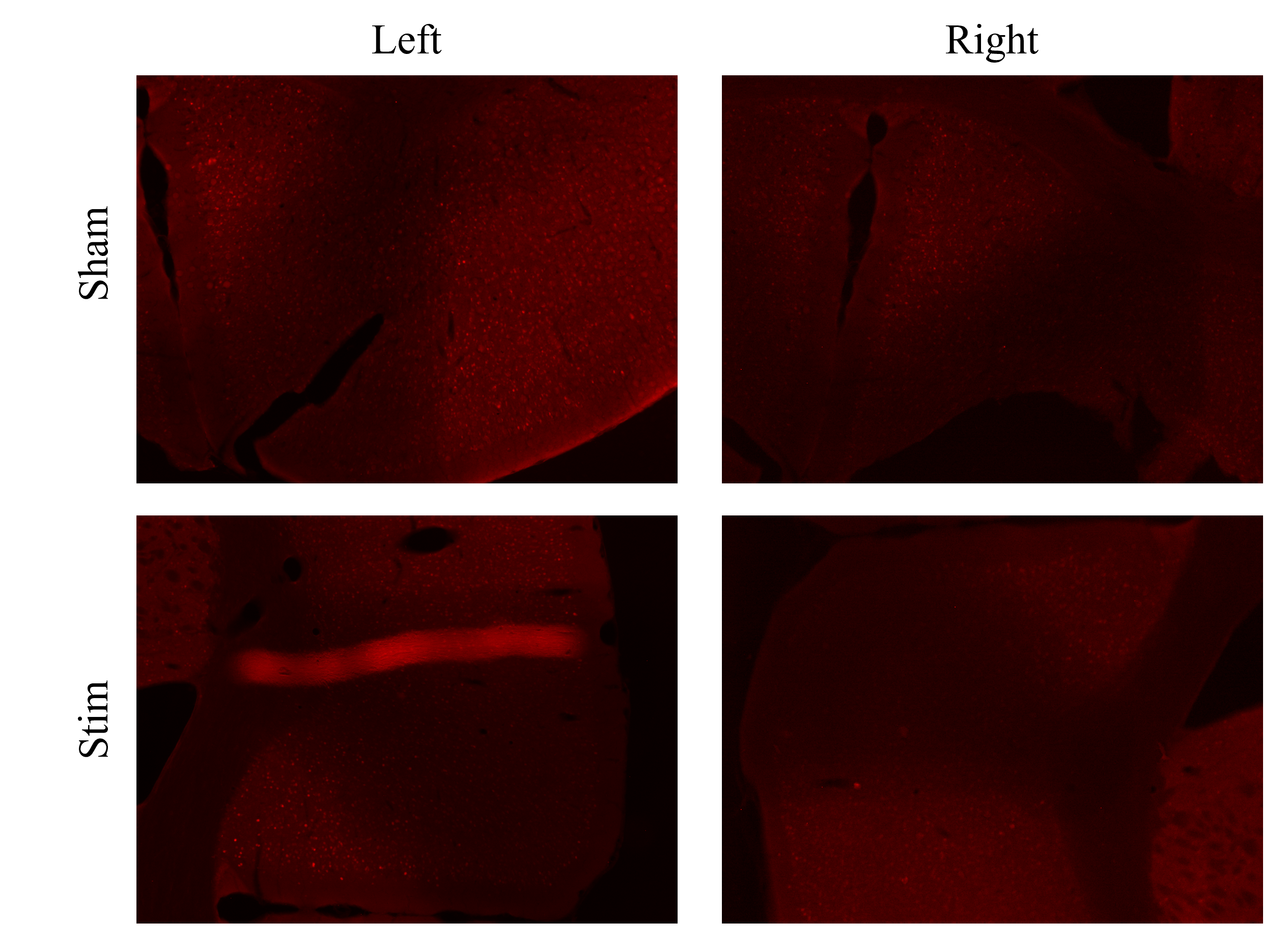


**Figure S9:** c-Fos staining (red) of left and right PMC in Sham (non-stimulated, control) and in Stim (stimulated) WT mice demonstrating high expression of active neurons in the stimulated left PMC. Magnification 4x.


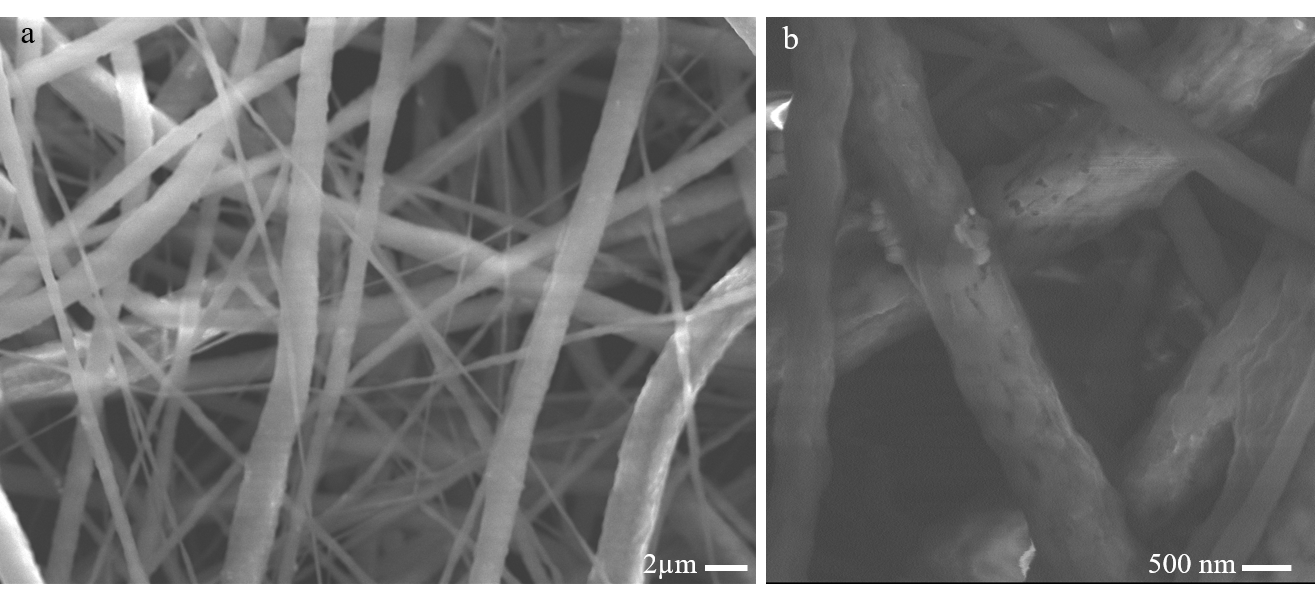


**Figure S10:** Micrograph of nanodiscs embedded in PVDF nanofibers, under a magnification of 3.5k (a) and 17k (b).
